# TRPV1 and TRPA1 Channels Are Both Involved Downstream of Histamine-Induced Itch

**DOI:** 10.3390/biom11081166

**Published:** 2021-08-06

**Authors:** Jenny Wilzopolski, Manfred Kietzmann, Santosh K. Mishra, Holger Stark, Wolfgang Bäumer, Kristine Rossbach

**Affiliations:** 1 Department of Pharmacology, Toxicology and Pharmacy, University of Veterinary Medicine Hannover, Foundation, 30559 Hannover, Germany; Manfred.Kietzmann@tiho-hannover.de (M.K.); rossbach@wdt.de (K.R.); 2 Department of Molecular Biomedical Sciences, College of Veterinary Medicine, North Carolina State University, Raleigh, NC 27607, USA; skmishra@ncsu.edu (S.K.M.); Wolfgang.Baeumer@fu-berlin.de (W.B.); 3 Department of Veterinary Medicine, Institute of Pharmacology and Toxicology, Freie Universität Berlin, 14195 Berlin, Germany; 4 Institute of Pharmaceutical and Medical Chemistry, Heinrich Heine University Düsseldorf, 40225 Duesseldorf, Germany; stark@hhu.de

**Keywords:** histamine, histamine H1 receptor, histamine H4 receptor, itch, signal transduction, TRPV1, TRPA1, dorsal root ganglion neurons (DRG), Ca^2+^-imaging

## Abstract

Two histamine receptor subtypes (HR), namely H1R and H4R, are involved in the transmission of histamine-induced itch as key components. Although exact downstream signaling mechanisms are still elusive, transient receptor potential (TRP) ion channels play important roles in the sensation of histaminergic and non-histaminergic itch. The aim of this study was to investigate the involvement of TRPV1 and TRPA1 channels in the transmission of histaminergic itch. The potential of TRPV1 and TRPA1 inhibitors to modulate H1R- and H4R-induced signal transmission was tested in a scratching assay in mice in vivo as well as via Ca^2+^ imaging of murine sensory dorsal root ganglia (DRG) neurons in vitro. TRPV1 inhibition led to a reduction of H1R- and H4R- induced itch, whereas TRPA1 inhibition reduced H4R- but not H1R-induced itch. TRPV1 and TRPA1 inhibition resulted in a reduced Ca^2+^ influx into sensory neurons in vitro. In conclusion, these results indicate that both channels, TRPV1 and TRPA1, are involved in the transmission of histamine-induced pruritus.

## 1. Introduction

Histamine is one of the most intensively studied mediators of itch. Histamine acts via four G protein-coupled receptors. Two of the four known histamine receptors (histamine H1 receptor (H1R) and histamine H4 receptor (H4R)) are involved in the induction of histamine-induced pruritus [1,2]. Additionally, the blockade of the histamine H3 receptor (H3R) seems to be involved in histamine-induced pruriception [2]. Although histamine has been known for almost 100 years to induce itch in humans, the exact signal transduction pathways are still not fully elucidated [3]. Key players in the signal transduction of itch are members of the transient receptor potential (TRP) family. Among the six subgroups of TRP channels in mammals, the transient receptor potential vanilloids 1 (TRPV1), TRPV3, TRPV4, ankyrin 1 (TRPA1), and melastin 8 (TRPM8) have been proposed to be involved in itch transduction [4]. Several groups have demonstrated that TRPV1 is important for the signal transduction of histamine-induced itch [2,5,6,7,8,9]. Histamine-induced pruritus is transmitted via specific mechano-insensitive C fibers [10]. Dorsal root ganglia (DRG), which contain the cell bodies of the sensory afferents, express all four histamine receptor subtypes [2,7,11]. The histamine-induced Ca^2+^ influx in DRG neurons is thought to be mediated via the H1R, H3R and H4R, and is related to capsaicin sensitivity [2,5,6,7,8,9]. Moreover, in mice treated with a TRPV1 blocker as well as in mice lacking the TRPV1 (TRPV1^−/−^) channel, the histamine-induced scratching behavior is reduced [9]. Furthermore, histamine enhances the production of 12-hydroxyeicosatetraenoic acid (12-HETE), a 12-lipoxygenase metabolite of arachidonic acid and an endogenous TRPV1 activator [9,12]. These results strongly indicate that histamine requires the activation of TRPV1 to excite sensory neurons and to induce itch. However, histamine induces a small increase of intracellular Ca^2+^ in about 10% of neurons of TRPV1-deficient mice. Additionally, some neurons of wild type mice responding to histamine are not capsaicin-sensitive, and histamine-induced scratching behaviors in TRPV1^−/−^ mice were decreased but not completely abolished, which suggests the involvement of other receptors in the itch response [2,4,9]. In addition, the different histamine receptors might use different downstream signaling pathways. Apart from TRPV1, sensory neurons show a strong expression of TRPA1. Both play crucial roles in detecting pruritogens and nociceptive stimuli [4]. 

This study was performed to investigate the involvement of TRPV1 and TRPA1 in histamine-induced itch, and to detect potential differences in the signaling pathways of the different histamine receptors. The role of TRPV1 and TRPA1 in histamine-induced pruritus as well as in histamine-induced Ca^2+^ increase in DRG neurons was analyzed after pharmacological blockade of both TRP channels. Additionally, histamine-induced itch was analyzed in vivo in TRPV1^−/−^ and TRPA1^−/−^ mice. To determine which mouse strain would be most suitable for our study, the scratching behavior following injection of a H4R-agonist of four different mouse strains was compared initially. 

## 2. Materials and Methods

### 2.1. Animals

Female BALB/c mice (BALB/cAnNCrl, (7 ± 1 weeks old, body weight 18 ± 1 g)), female CD-1 mice (Crl:CD-1, (7 ± 1 weeks old, body weight 27 ± 1 g)), and female NMRI mice (Crl:NMRI, (7 ± 1 weeks old, body weight 20 ± 1 g)) were obtained from Charles River (Sulzfeld, Germany, or Raleigh, NC, USA). Female and male C57/BL/6 mice (C57/BL/6J, (7 ± 1 weeks old, body weight 20 ± 1 g)), female and male TRPV1^−/−^ mice (B6.129X1-Trpv1^tm1Jul^/J, (7 ± 1 weeks old, body weight 20 ± 1 g)) and female and male TRPA1^−/−^ mice (B6;129P-Trpa1^tm1Kykw^/J, (7 ± 1 weeks old, body weight 20 ± 1 g)) were obtained from the Jackson Laboratory (Bar Harbor, ME, USA). All animals were kept in groups of four or six mice per cage with a 12 h light/dark cycle at 22 °C. Water and a standard diet (Altromin 1824/LabDiet 5001) were provided ad libitum. The animal experiments have been ethically approved by the LAVES institute, Oldenburg, Germany (AZ 33.12-42502-04-16/2213, approval date: 28.09.16) and by the North Carolina State University Animal Care and Use Committee (IACUC Protocol No. 16-154-B and 16-038-B (1)). 

### 2.2. Reagents

Histamine dihydrochloride and capsaicin (TRPV1 agonist) were purchased from Sigma-Aldrich (Steinheim, Germany). The 4-methylhistamine dihydrochloride (4-MH; H2R /H4R agonist), and histamine trifluoromethyl toluidine dimaleate (HTMT; H1R/H2R agonist) were obtained from Tocris Bioscience (Bristol, UK). ST-1006 (H4R agonist) and JNJ-7777120 (H4R antagonist) were synthesized and provided by Prof. Dr. H. Stark (Heinrich-Heine-Universität, Düsseldorf, Germany) [13,14]. Allylisothiocyanate (AITC; TRPA1 agonist) and ruthenium red (TRP channel inhibitor) were purchased from Acros Organics, Morris Plaines, NJ, USA. Capsazepine (TRPV1 inhibitor), SB366791 (TRPV1 inhibitor) and HC-030031 (TRPA1 inhibitor) were obtained from Cayman Chemicals (Ann Arbor, MI, USA). Diphenhydramine hydrochloride (H1R antagonist) was purchased from West Ward Pharmaceuticals (Eatontown, NJ, USA).

### 2.3. Evaluation of Scratching Behavior

Scratching behavior was analyzed as an indicator of pruritus. Mice were acclimatized to their environment for 2 weeks before the experiments. Mice were randomly allocated into different treatment groups (*n* = 6 (H4R-induced scratching behavior in four different mouse strains) and *n* = 9 (effects of TRP channels on histamine induces itch) for each group). Group sizes were determined from a power analysis with the software G*Power 3.1.9.2. A co-worker blinded to the experimental protocol randomized animals into these groups. One day before each experiment, the rostral part of the neck was clipped with electric clippers. To measure strain dependent differences in the response to H4R-induced itch, the H4R agonist 4-MH (500 nmol/L NaCl) was injected intradermally (i.d.) into the neck. Application of 50 µL sterile saline (0.9% NaCl) was used as vehicle control. The strain with the most pronounced scratching response was used for further experiments. 

HC-030031 (60 mg/kg), Capsazepine (6 mg/kg) or SB366791 (0.5 mg/kg) were given intraperitoneal (i.p.; 200 µL) 45 min before injection of histamine (25 nmol/L), HTMT (100 nmol/L), 4-MH (50 nmol/L) or ST-1006 (50 nmol/L). For the experiments in knockout mice, histamine (800 nmol/L), 4-MH (500 nmol/L) or ST-1006 (100 nmol/L) were injected intradermally. After injection of the histamine receptor agonists, mice were placed in observation chambers and recorded on video for 30 min. Afterwards, scratching bouts were analyzed in a blinded manner. According to Kuraishi et al. (1995), a scratching bout was defined as a series of scratching movements by a hind paw in the area around the injection site until the paw was licked by the mouse or placed on the ground [15].

### 2.4. Isolation of DRG Neurons

Isolation of DRG neurons was performed according to Rossbach et al. et al. (2011) [2]. To collect DRGs, mice were deeply anaesthetized with CO_2_ and then exsanguinated. Then, 15–20 DRGs were collected along the whole opened vertebral column. DRGs were enzymatically digested in dispase II (2.5 mg/mL; Stemcell Technologies, Vancouver, Canada) and collagenase from *Clostridium histolyticum* (2.5 mg/mL; Sigma-Aldrich, St. Louis, MO, USA) dissolved in Ca^2+^- and Mg^2+^-free Hank’s Buffered Salt solution (Thermo Fisher Scientific, Fair Lawn, NJ, USA) for a total of 60 min at 37 °C. Neurons were dissociated every 30 min. with pasteur pipettes.

The cells were washed with DMEM medium (Corning, Manassas, VA, USA) containing 10% FBS (Mediatech Inc., Manassas, VA, USA) and 1% Penicillin/Streptomycin (Pen/Strep; Corning, Manassas, VA, USA) by centrifugation and resuspended in 160 µL media. Then, 20 µL of the cell suspension were transferred to poly-l-lysine hydrobromide (0.1 mg/mL; Sigma-Aldrich, St. Louis, MO, USA) and laminin (0.1 mg/mL; Sigma-Aldrich, St. Louis, MO, USA) coated glass coverslips (18 mm, round; Warner Instruments; Hamden, CT, USA) and incubated for 2 h at 37 °C and then flooded with a larger volume of DMEM/10% FBS/1% Pen/Strep. Cells were incubated at 37 °C overnight until measurements were performed less than 24 h later. 

### 2.5. Ca^2+^-Imaging

Changes in intracellular free Ca^2+^ concentration in single cells were measured by digital microscopy connected to equipment for ratiometric recording of single cells as described previously [2]. The cultured neurons were loaded with 8 µmol/L Fura-2-acetylmethylester (Biotium, Freemont, CA, USA) in DMEM media, protected from light, for 40 min at 37 °C. 

The neuron-covered coverslip was inserted into the chamber (Warner Instruments, Hamden, CT, USA) of the imaging system and constantly perfused with 36 °C Lockes’ buffer containing (mmol/L) 136 NaCl, 5.6 KCl, 1.2 MgCl_2_, 2.2 CaCl_2_, 1.2 NaH_2_Po_4_, 14.3 NaHCO_3_, 10 d-Glucose (pH 7.3–7.4). The cells were monitored on an inverted microscope (Nikon TE200, Nikon Instruments, Melville, NY, USA) by sequential excitation at 340 and 380 nm. Fluorescence intensities at both wavelengths were measured every 500 ms by using a camera attached to the Lambda LS lamp and a Lambda optical filter changer. Images were obtained using PC-based software, and the Fura-2 ratio (F340/380) was calculated (NIS-Elements AR 5.02.01; Nikon Instruments, Melville, NY, USA). Regions of interest (ROIs) were defined around each neuron according to their neuron typical morphology. DRG neurons from CD-1 mice were exposed to control solution (Lockes’ buffer) followed by 4-MH (0.1 mmol/L), ST-1006 (0.1 mmol/L), HTMT (0.1 mmol/L) or histamine (1 mmol/L). For testing the direct effects of the TRP inhibitors, ruthenium red, HC-033301 or SB366791 were administered 15–30 s prior to the stimulus in a concentration of 1 or 10 µmol/L. The histamine receptor antagonists diphenhydramine (H1R) and JNJ-7777120 (H4R) were applied 15–30 s prior to the stimulus in a concentration of 10 µmol/L to test the specificity of the histamine receptor agonists used. To functionally determine to which extent H4R- and histamine-positive cells reacted to the TRP channel agonist, DRG neurons of CD-1, C56BL/6 and BALB/c mice were exposed subsequently to 4-MH (0.1 mmol/L), histamine (1 mmol/L), AITC (1 µmol/L) and capsaicin (1 µmol/L). This last experimental setting aimed to gain more information about strain differences in the reaction profile of the neurons to these substances. At the end of each measurement, potassium chloride (KCl; 150 mmol/L) was applied to confirm the viability of the neurons. The cells were washed with fresh buffer for two min after each stimulus to recover cells prior to the addition of the next stimulus. 

The 340/380 ratio directly reflects the Ca^2+^ influx into the sensory neurons upon simulation. All fura-2 measurements were normalized to the resting baseline ratio F340/F380. If the ratio value increased by more than 10% of the resting level after stimuli application, the neurons were considered as activated by the substance tested. Only the cells that reacted to KCl at the end of each measurement were included into analysis.

### 2.6. Statistics

All figures for the in vivo data are presented as scatter-dot plots with median ± SD. Data of the in vivo experiments did not follow normal distribution, thus significant differences were assessed with the nonparametric Mann–Whitney U test compared to the control group. Differences in scratching response over time were analyzed with a two-way ANOVA followed by Sidak’s multiple comparisons test. Significant differences between calcium peaks induced by the test drugs with or without inhibitor pretreatment were analyzed by the Fisher’s exact test. A *p* value of less than 0.05 was regarded as statistically significant. For the statistical analysis, the program Graph Pad prism version 7 (GraphPad software, Inc., San Diego, CA, USA) was used. 

## 3. Results

**Figure 1:**
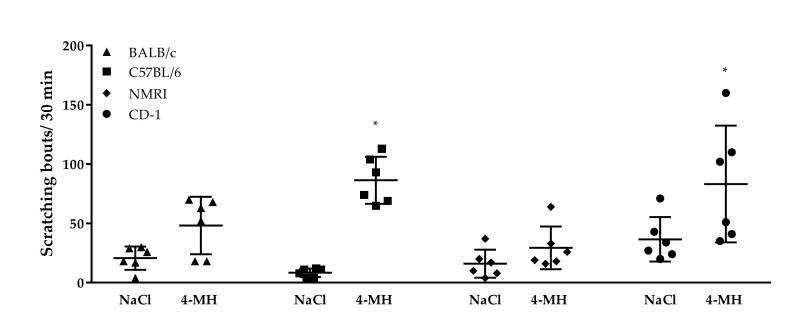
Strain differences in sensitivity to itch induced by 500 nmol/L 4-methylhistamine. Both BALB/c and NMRI mice did not show a significant increase in scratching response to intradermal 4-MH injection, whereas 4-MH induced a significant scratching response in C57BL/6 and CD-1 mice. Observation time: 30 min. Results are shown as scatter-dot plot with mean ± SD. *n* = 6, * *p* < 0.05 (Mann–Whitney U test) = significantly different from vehicle control (saline) injection.

We first examined which of the four mouse strains (BALB/c, C57BL/6, CD-1, NMRI) showed the most pronounced scratching response to a H4R-agonist (4-MH). The 4-MH at a concentration of 500 nmol/L did not elicit a robust scratching behavior in BALB/C or NMRI mice. A significant increased number of scratching bouts compared to vehicle injection was observed in C57BL/6 and CD-1 mice (Figure 1). In line with Inagaki et al. (2001) [16] and Bell et al. (2001) [1], who identified CD-1 mice reacting with the highest scratching response after intradermal injection of histamine, further experiments in this study were conducted in CD-1 mice. 

**Figure 2:**
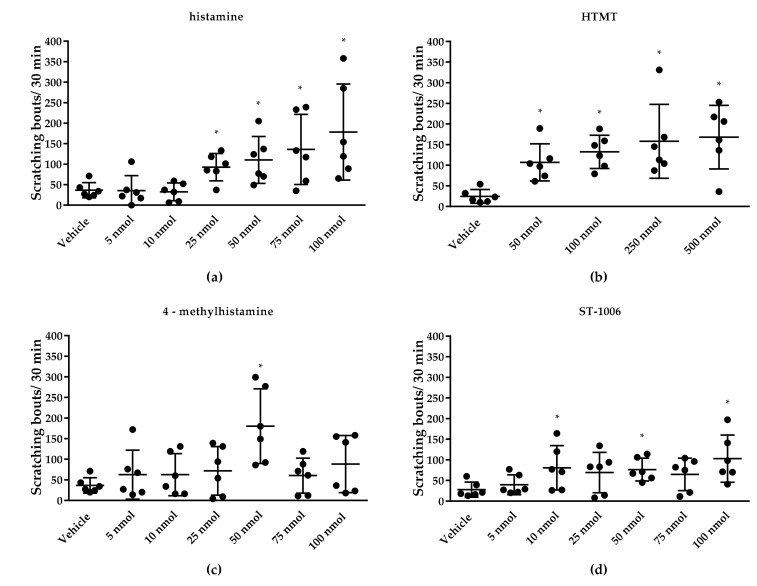
Concentration-dependent induction of itch by different histamine receptor agonists after intradermal application (50 µL) in CD-1 mice. (**a**) Histamine (all HR) induced a significant scratching response at concentrations of 25–100 nmol/L. (**b**) HTMT (H1R/H2R) induced a significant scratching response at 50–500 nmol/L. (**c**) 4-MH (H2R/H4R) induced a significant scratching response at 50 nmol/L. (**d**) ST-1006 (H4R) induced a significant scratching response at 10, 50 and 100 nmol/L. * *p* < 0.05 (Mann–Whitney U test) = significantly different from vehicle control (saline) injection.

**Figure 3:**
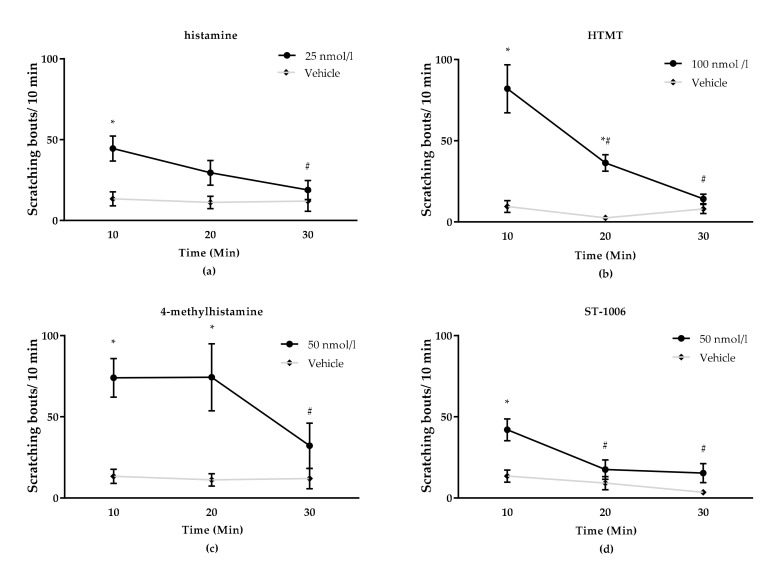
Scratching response time course over 30 min after intradermal injection (50 µL) of histamine receptor agonists in CD-1 mice. All agonists induced a significantly increased scratching response in the first 10 min after application. HTMT- and 4-MH-induced itch returned to baseline level (vehicle control = 0.9% NaCl) at approximately 30 min after intradermal injection. For histamine- and ST-1006-induced itch, no significant difference was seen 20 min after injection. (**a**) Scratching response after 25 nmol/L histamine (all HR) injection. (**b**) Scratching response after 100 nmol/L HTMT (H1R/H2R) injection. (**c**) Scratching response after 4-MH (H2R/H4R) 50 nmol/L injection. (**d**) Scratching response after ST-1006 (H4R) 50 nmol/L injection. Results are presented as median ± SD. Two-way ANOVA: * *p* < 0.05 (factor treatment) = significantly different from vehicle (saline) injection, # *p* < 0.05 (factor time) = scratching response to pruritogen significantly different from 10 min timepoint.

Second, we determined the dose response for the histamine receptor agonists in CD-1 mice. All agonists used in this study were tested in CD-1 mice at dosages from 5 to 100 nmol/L (histamine, 4-MH, ST-1006) or 50 to 500 nmol/L (HTMT) (Figure 2). Doses that elicited a robust scratching behavior in CD-1 mice were used for subsequent experiments. The following doses were chosen: histamine: 25 nmol/L, 4-MH: 50 nmol/L, ST-1006: 50 nmol/L and HTMT: 100 nmol/L (Figure 3). 

### 3.1. Effect of TRP Channels on Histamine-Induced Pruritus

**Figure 4:**
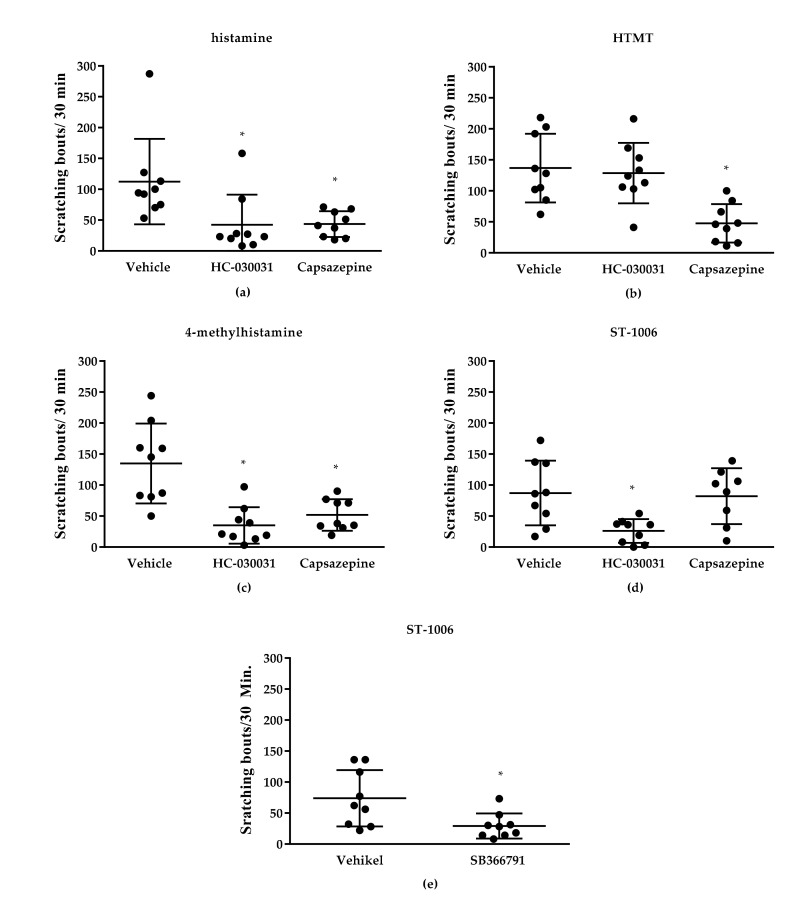
Influence of the TRPA1 inhibitor HC-030031 and the TRPV1 inhibitor capsazepine or SB366701 on histamine-induced itch in CD-1 mice. (**a**) Histamine (all HR)-induced pruritus (25 nmol/L, i.d.) was significantly reduced by both tested inhibitors. (**b**) HTMT (H1R/H2R)-induced pruritus (100 nmol/L, i.d.) was significantly reduced by capsazepine. (**c**) 4-MH (H2R/H4R)-induced pruritus (50 nmol/L, i.d.) was significantly reduced by both tested inhibitors. (**d**) ST-1006 (H4R)-induced pruritus (50 nmol/L, i.d.) was significantly reduced by HC-030031. (**e**) ST-1004 (H4R)-induced pruritus (50 nmol/L i.d.) was significantly reduced by SB366791. Observation time: 30 min. Results are displayed as scatter-dot plots with median ± SD. *n* = 9 (*n* = 8 for ST-1006 + capsazepine—exclusion of one mouse due to stereotypical behavior during video monitoring). * *p* < 0.05 (Mann–Whitney U test) = significantly different from vehicle. Dose per 200 µL i.p. injection of inhibitors: HC-030031: 60 mg/kg, capsazepine: 6 mg/kg, SB366791: 0.5 mg/kg, vehicle = 10% DMSO.

**Figure 5:**
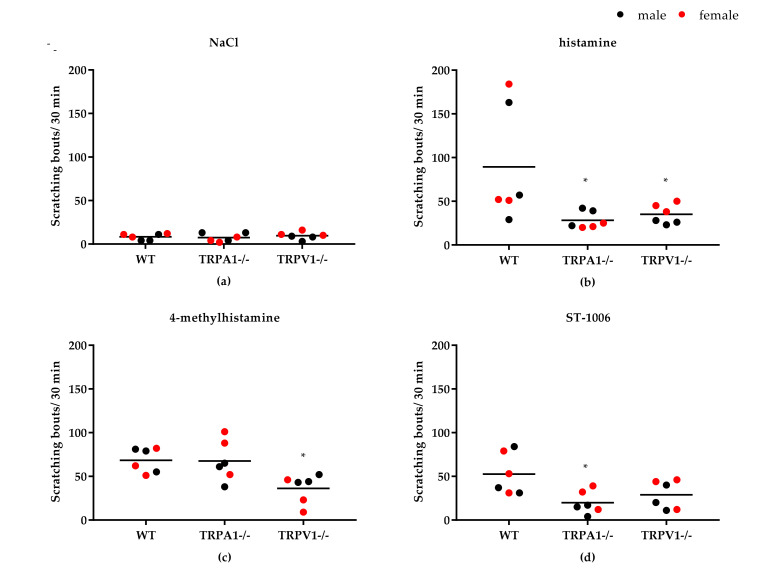
Histamine-induced itch in TRPV1^−/−^ and TRPA^−/−^ mice compared to wild type mice (C57BL/6; WT). (**a**) No significant scratching response after 0.9% NaCl (vehicle control) injection (50 µL, i.d.) in all three mouse strains. (**b**) Both TRPV1^−/−^ and TRPA^−/−^ mice showed a significantly lower scratching response to histamine (all HR) injection (800 nmol/L, i.d.) compared to wild type mice. (**c**) TRPV1^−/−^ showed a significantly lower response to 4-MH (H2R/H4R) injection (500 nmol/L, i.d.) compared to wild type mice. (**d**) TRPA^−/−^ mice showed a significantly lower scratching response to ST-1006 (H4R) injection (100 nmol/L, i.d.). Observation time: 30 min. Results displayed as scatter-dot plots with median. *n* = 6 per group (*n* = 3 per sex). * *p* < 0.05 (Mann–Whitney U test) = significantly different from wild type.

Pretreatment with the TRPV1 inhibitor capsazepine (6 mg/kg, i.p.) reduced scratching behavior induced by histamine, by the H1R/H2R agonist HTMT and by the H2R/H4R agonist 4-MH (Figure 4). However, itch induced by the selective H4R agonist ST-1006 could not be attenuated with capsazepine (Figure 4). An additional experiment with the TRPV1 inhibitor SB366791 (0.5 mg/kg, i.p.) revealed that ST-1006-induced itch could significantly be decreased by this inhibitor (Figure 4). The TRPA1 inhibitor HC-030031 (60 mg/kg, i.p.) reduced histamine-induced itch as well as itch induced by the H4R agonists 4-MH and ST-1006. H1R-induced itch in contrast was not affected by the TRPA1 inhibitor (Figure 4). Inhibitor concentrations were determined according to literature [9,17,18]. In TRPV1^−/−^ and TRPA1^−/−^ mice, scratching behavior induced by histamine or H4R agonists was significantly lower compared to wild type mice (Figure 5).

### 3.2. Effect of TRP Channels on Histamine-Induced Intracellular Ca^2+^-Increase

**Figure 6:**
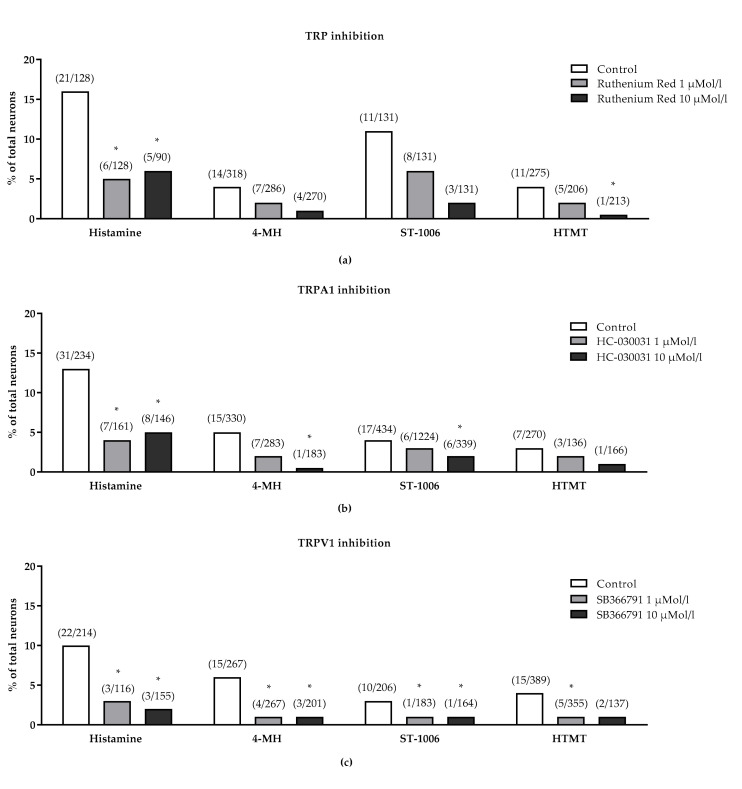
Influence of different TRP channel inhibitors on histamine-induced intracellular Ca^2+^-increase (change in 340/380 nm fluorescence intensity ratio) in DRG neurons of CD-1 mice. Neurons were stimulated with histamine (1 mmol/L), 4-MH (100 µmol/L), ST-1006 (100 µmol/L) or HTMT (100 µmol/L). (**a**) The TRP channel blocker ruthenium red concentration dependently inhibited the neuronal response of all histamine ligands tested. (**b**) The TRPA1 channel blocker HC-030031 concentration dependently inhibited the neuronal response to histamine, 4-MH and ST-1006. (**c**) The TRPV1 channel blocker SB366791 concentration dependently inhibited the neuronal response of all histamine ligands tested. Total numbers of reactive cells and their percentage to the total numbers of cells were examined (KCl 150 mmol/L as positive control). * *p* < 0.05 (Fisher’s exact test) = significantly different from control; DRG neurons were collected from n = 4 mice per inhibitor group.

To determine whether the TRPV1 or TRPA1 channel is involved in H4R-induced neuronal excitation, cells were pre-incubated with the TRPV1-inhibitor SB366791 or the TRPA1 inhibitor HC-030031. Both inhibitors dose dependently reduced the intracellular Ca^2+^-increase after stimulation with histamine, 4-MH and ST-1006. Furthermore, SB366971 also reduced the HTMT-induced intracellular Ca^2+^-increase. In addition, the TRP channel blocker ruthenium red concentration dependently inhibited the intracellular Ca^2+^-increase after stimulation with histamine, HTMT, 4-MH and ST-1006 (Figure 6).

To evaluate the specificity of the HR agonists, we analyzed the neuronal response to these in combination with selective HR antagonists. Intracellular Ca^2+^-increase induced by the dual H2R/H4R agonist 4-MH (9/196 cells, 5%) as well as by the H4R agonist ST-1006 (9/166, 5%) was blocked by the H4R antagonist JNJ-7777120 (4-MH 1/196, 0.5% and ST-1006 0/166, 0%). The Ca^2+^-increase induced by the dual H1R/H2R agonist HTMT (7/113, 6%) could be blocked with the specific H1R antagonist diphenhydramine (1/113, 1%).

### 3.3. Role of TRPV1 and TRPA1 on 4-MH-Induced Ca^2+^-Increase in DRG Neurons in Three Different Mouse Strains 

**Figure 7:**
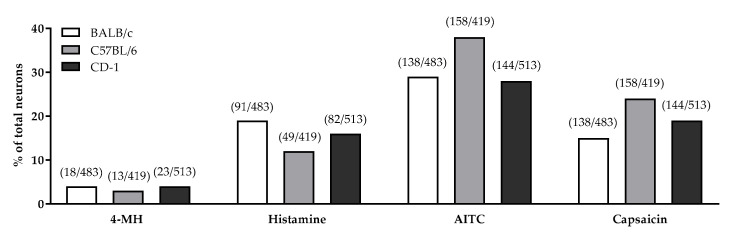
Drug-induced increase in intracellular Ca^2+^ (change in 340/380 nm fluorescence intensity ratio) in DRG neurons from BALB/c, C57BL/6 and CD-1 mice in response to 4-MH (100 µmol/L), histamine (1 mmol/L), AITC (1 µmol/L), and capsaicin (1 µmol/L). Total numbers of reactive cells and their percentage of total cell number were examined (KCl 150 mmol/L as positive control).

**Figure 8:**
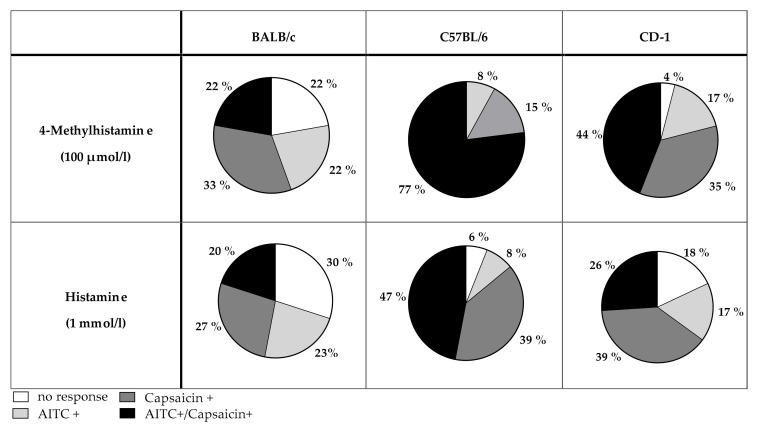
Differences in the reaction of 4-MH- and histamine-sensitive DRG neurons to TRPA1 (AITC, 1 µmol/L) and TRPV1 (capsaicin, 1 µmol/L) agonists in BALB/c, C57BL/6 and CD-1 mice. Neurons were classified into four groups: 1. Neurons not responding to any TRP channel agonist (=no response, white). 2. Neurons responding to capsaicin only (=capsaicin +, light grey). 3. Neurons responding to AITC only (=AITC+, dark grey). 4. Neurons responding to both AITC and capsaicin (=AITC+/capsaicin+, black). The pie charts show percentage values related to the total number of cells examined. Significantly more 4-MH neurons of C57BL/6 reacted to both AITC and capsaicin compared to BALB/c (*p* < 0.04) and CD-1 (*p* < 0.08). In alignment, more histamine-sensitive neurons of C57BL/6 reacted to both AITC and capsaicin compared to BALB/c (*p* < 0.02) and CD-1 mice (*p* < 0.002). Statistical significances were calculated with the Fisher’s exact test, and *p* values < 0.05 were considered statistically significant.

In all mouse strains tested (CD-1, BALB/c and C57BL/6 mice) 4-MH induced a Ca^2+^ increase in 3–4% of DRG neurons (Figure 7). In contrast, histamine activated 19% of DRG neurons isolated from BALB/c, 16% of neurons isolated from CD-1 mice, and 12% of neurons isolated from C57BL/6. After application of the TRPA1 agonist AITC, significantly more neurons from C57BL/6 mice (38%) were activated compared to neurons isolated from CD-1 (28%) or BALB/c mice (29%). After capsaicin (TRPV1 agonist) stimulation, fewer neurons obtained from BALB/c mice (15%) reacted with an intracellular Ca^2+^-increase compared to neurons of CD-1 (19%) and C57BL/6 mice (24%). The majority of 4-MH positive cells isolated from CD-1 (44%) and C57BL/6 mice (77%) were sensitive to both AITC and capsaicin (Figure 8). Most of the 4-MH-sensitive neurons obtained from BALB/c mice responded to the TRPV1 agonist capsaicin (33%) in contrast and to a lesser extent to AITC (22%). Only 22% of 4-MH-sensitive neurons of BALB/c mice responded to both capsaicin and AITC. 

## 4. Discussion

Among the broad variety of pruritogens, histamine is one of the most comprehensively studied itch mediators. Histamine acts via four G-protein coupled receptors (H1-4R). In addition to the H1R, the H4R seems to be the most relevant histamine receptor in the transmission of histamine-induced itch [1,2,19]. Antagonists targeting the H4R are effective in reducing histamine- and allergen-induced itch in rodents and humans, and are thus discussed as new therapeutic options for the treatment of pruritic skin diseases [20,21,22,23,24]. However, the exact signal transduction mechanisms of histamine-induced itch—especially via the H4R—are still not fully understood. Previous studies stated that only the TRPV1 channel is involved in the signaling mechanisms of histamine-induced itch [4,8,9,25]. 

In this study, we demonstrated for the first time that in addition to the TRPV1, the TRPA1 channel also seems to be associated with histamine-induced itch transduction via the H4R. This is in contrast to the published consensus that the TRPA1 channel is not involved in histamine-induced itch [4,25]. Various studies demonstrated a reduced scratching behavior in TRPV1^−/−^ mice in response to histaminergic and non-histaminergic pruritogens, whereas a reduced scratching response was seen in TRPA1^−/−^ mice in response to non-histaminergic pruritogens compared to wild type mice [9,25,26]. Supporting the in vivo data of our study, which showed an involvement of the TRPA1 channel in H4R-signaling, a lower number of DRG neurons obtained from CD-1 mice responded to histamine or the H4R agonists (4-MH and ST-1006) after pre-incubation with HC-030031. These findings are again in contrast to a study by Jian et al. (2016) [6], in which HC-030031 could not block the Ca^+^ influx in DRG neurons obtained from 4 weeks old C57BL/6 mice. In this study Ca influx was induced by the H4R agonist immepip [6]. However, immepip has a higher affinity to the H3R (p*k*_i_ = 9.3) than to H4R (p*k*_i_ = 7.7) [27]. Thus, an involvement of the H3R cannot be precluded in these data [2]. The utilization of different H4R agonists in this and other published studies, with their specific, possibly unknown off-target effects, together with the varying mouse strains, makes the interstudy comparison of results difficult [28]. 

Indeed, no study could ever completely inhibit histamine-induced itch with TRPV1 inhibitors alone or in TRPV1^−/−^ mice [6,9]. This implicates a possible involvement of other TRP channels in histamine signaling, especially the TRPA1 channel. TRPA1 in fact is co-expressed within a subpopulation of TRPV1-expressing sensory neurons (30%) [29]. Further functional in vitro studies showed subpopulations of histamine-sensitive trigeminal ganglion neurons and DRG neurons, which were sensitive to the TRPA1 agonist AITC and/or the TRPV1 agonist capsaicin [30,31]. In trigeminal ganglion neurons, 70% of histamine-responding cells reacted to capsaicin and 39% to AITC [30]. Although not mentioned, an overlap between these TRPA1- or TRPV1-responsive subpopulations seems inevitable. In a study by Zhang (2015) [31], 41% of histamine-positive DRG neurons of neonatal C57BL/6 mice were sensitive for both AITC and capsaicin. Similar results were found in the present study: depending on the mouse strain, we evaluated that 22–77% of neurons reacting to 4-MH also reacted to AITC and capsaicin (Figure 8). In line with this, the TRPA1/TRPV1 inhibitor ruthenium red significantly reduced the intracellular Ca^2+^ increase after application of histamine 4-MH and ST-1006. In a pilot experiment in TRPV1^−/−^/TRPA1^−/−^ mice (*n* = 3 mice), the H4R-induced scratching response was nearly diminished to a baseline level (data not shown). A possible link between histamine and TRPA1 signaling might be thymic stromal lymphopoietin (TSLP), a known progressor of allergic diseases. TSLP is released by keratinocytes after stimulation with histamine [32]. The interaction of keratinocytes and neurons in the onset and progression of itch has already been addressed [33]. TSLP directly activates sensory neurons and promotes itch via the TRPA1 channel on these cells [32]. TSLP release by keratinocytes is thought to be mediated via the H4R both in human and murine keratinocytes [34]. Briefly, these data implicate a possible link between TRPV1, TRPA1 and HR in histamine signal transduction.

Interestingly, Ru et al. (2017) [35] presented data in a skin-nerve preparation of TRPV1^−/−^/TRPA1^−/−^ mice that argue against an involvement of TRP channels in the onset of histamine- and chloroquine-induced itch. In this study, the response of itch-specific peripheral C-fibers of these knockout mice compared to wild type mice did not differ after pruritogen application. According to the authors, these data are not necessarily contradictive to an involvement of TRP channels in itch transmission [35]; their involvement might rather be associated with the inhibition of the inflammatory response and production of pruritogens than with a direct regulation of action potential generation at nerve terminals [35]. In an IL-13-induced mouse model of atopic dermatitis, blocking the TRPA1 led to a reduction of the scratching response [36]. As the H4R activates signaling pathways to induce cytokine and chemokine production, for example of IL-13 and RANTES (Regulated upon Activation Normal T cell Expressed and Secreted) in mast cells, the involvement of TRPA1 channels in histamine—especially H4R-induced itch—cannot be excluded [37].

As a study limitation, it has to be considered that results obtained in TRPA1^−/−^ and TRPV1^−/−^ mice were not completely congruent with the results obtained with pharmacological blockade of the TRPA1 or TRPV1 channels in CD-1 mice and vice versa. These heterogeneous and partially contradictory results obtained in knockout mice compared to the chemical inhibition of the TRP channels clearly need to be considered as a limitation of this study. They emphasize the need for further investigation of to which extent the genotype affects the sensation of pruritogens and their signal transmission. Although using two different TRPV1 inhibitors for the in vivo and the in vitro part of this study can be considered as a limitation, the results obtained in both setups are reasonably consistent. Off-target effects of all chemical compounds used, as well as molecular or cellular compensation mechanisms, which may occur in knockout mice, might be possible pitfalls. The theory of compensation mechanisms in connection with TRP channels, for example, has been reported by Petrus et al. (2007) [38]. TRPA1 is known to be involved in noxious mechano- and cold thermosensation. Nevertheless, in the study by Petrus et al. (2007) [38], TRPA1^−/−^ mice showed normal hyperalgesia, whereas a specific TRPA1 antagonist could reduce cinnamaldehyde-induced nociception in vivo. 

A specificity study on H4R agonists showed that effects (reduced IL-12p70 secretion from monocytes) caused by 4-methylhistamine could not completely be diminished by the selective H4R antagonist JNJ7777120, while ST-1006-induced effects could be blocked completely [39]. Yet, taking into consideration that 4-MH is a H2R/H4R agonist, the involvement of the H2R in itch has, except for one study with *n* = 3 mice, not been investigated [1]. Thus, further investigation is needed to determine the biochemical reasons for the different results seen for 4-MH and ST-1006 in vivo and in vitro. Furthermore, some H4R ligands exhibit a functional selectivity on the H4R by stabilizing multiple ligand-specific receptor conformations [40,41]. Although being an H4R antagonist, JNJ7777120 exhibited context-dependent stimulatory effects on the H4R, for example [42]. 

As already mentioned, mouse strain differences complicate the interpretation of the results presented here. As not every animal species or strain is suitable for every existing experimental set up, picking the appropriate mouse (animal) model for an investigation is of great importance to generate significant and ideally translational data [43,44,45]. As Inagaki et al. (2001) [16] pointed out, major strain-specific differences exist in the scratching response to various pruritogens such as histamine and serotonin. They identified the inbred mouse strain C57BL/6 and the outbred strain CD-1 (ICR) as the most susceptible strains to local histamine application. CD-1 mice reacted twice as intensively to histamine injection (50 nmol/L, i.d.) compared to C57BL/6 mice. Additionally, Bell et al. (2004) [1] mentioned CD-1 mice reacting 30 times more sensitively to histamine than BALB/c mice. In our study, a comparable effect could be shown for H4R agonist (4-MH)-induced itch for the first time. Both CD-1 and C57BL/6 showed a higher scratching response than NMRI and BALB/c mice. Until now, the underlying mechanisms for this difference in response to H4R-stimulation has not been examined. We hypothesized that receptors and ion channels involved in itch induction/transduction might be expressed in different quantities or combinations in the various strains. Our findings to 4-MH sensitivity of 3–4% of DRG neurons in the examined strains are in line with already published data (3–10% sensitivity to H4R agonists; [2,6]). In both CD-1 and C57BL/6 DRG neurons, the majority of 4-MH-sensitive neurons were both AITC and capsaicin responsive, whereas the majority of 4-MH-sensitive BALB/c DRG neurons were only capsaicin responsive. Interestingly, fewer neurons were histamine-sensitive in C57BL/6 (12%) compared to the other strains investigated (16–19%). Still, these results are consistent with values found in literature (11–16%; [5,7]). Within this population, in C57BL/6 mice, more cells reacted to AITC or both AITC and capsaicin stimulation than in the other strains examined. Possibly this difference compensates for the smaller amount of histamine-sensitive neurons. As these results only represent the functional properties of these cells, further studies should possibly address the receptor repertoire on these cells, for example, on the mRNA level. Based on the present data, there is no final explanation for the underlying mechanisms of the strain-specific differences in sensitivity to histamine or H4R agonists. Attention also needs to be given to other cells of the organism, which may be involved in the onset of pruritus, i.e., keratinocytes or mast cells [37,46]. Itch transduction is a complex interaction of receptors, second messengers, and other effector molecules in a variety of cells (for review see Cevikbas and Lerner, 2020 [47]).

A further aspect to be considered is the use of female mice in chemical TRP inhibition experiments, whereas for the experiments with the TRP knockout mice both sexes were used. Depending on the mouse genotype, female mice have been shown to be more sensitive to itch stimuli than male mice [44,48]. Evaluation of sex-specific differences was not part of this study; however, due to the low number of animals used (*n* = 3 per sex) and the absence of statistically significant differences between male and female mice, we decided to pool both sexes. Generally speaking, it would be best practice to use both sexes in each experimental set up, which in turn might increase the number of animals used in these studies and challenge the aspiration to reduce the number of laboratory animals used in research according to the 3R principle by Russel and Burch [49]. 

Remarkably in the in vitro part of this study, compared to 34–78% in literature, only 15–24% (Figure 8) of the total examined neurons reacted to the TRPV1 agonist capsaicin [2,5,6,9,26]. A physical and functional interaction between both TRPV1 and TRPA1 channels is well characterized [50,51,52,53]. As already discussed, TRPA1- and TRPV1-specific evoked responses undergo functional cross-desensitization in vivo and in vitro [50,54]. Consequently, cells activated by the application of the TRPA1 agonist AITC will respond in a less pronounced way to a subsequent treatment with the TRPV1 agonist capsaicin or vice versa. 

## 5. Conclusions

In conclusion, this study presents in vivo and in vitro evidence that in addition to the TRPV1, the TRPA1 channel also is responsible for histamine-induced itch transmission in mice. Furthermore, downstream signaling pathways of the H1R and the H4R seem to be different. Further experiments need to be conducted to determine the crosstalk between TRP channels and histamine receptors, and the subsequent signaling cascade. 

## Data Availability

Full data sets are available upon request from the corresponding author.
